# Cellular Integrin α5β1 and Exosomal ADAM17 Mediate the Binding and Uptake of Exosomes Produced by Colorectal Carcinoma Cells

**DOI:** 10.3390/ijms22189938

**Published:** 2021-09-14

**Authors:** Beatriz Cardeñes, Irene Clares, Víctor Toribio, Lucía Pascual, Soraya López-Martín, Alvaro Torres-Gomez, Ricardo Sainz de la Cuesta, Esther M. Lafuente, Manuel López-Cabrera, María Yáñez-Mó, Carlos Cabañas

**Affiliations:** 1 Centre for Molecular Biology “Severo Ochoa” (CSIC-UAM), Cell-Cell Communication & Inflammation Unit, 28049 Madrid, Spain; bea_car@hotmail.com (B.C.); ireneclares@gmail.com (I.C.); victor.toribio@estudiante.uam.es (V.T.); lucia.pascual@cbm.csic.es (L.P.); slopez@cbm.csic.es (S.L.-M.); mlcabrera@cbm.csic.es (M.L.-C.); maria.yannez@uam.es (M.Y.-M.); 2 Department of Molecular Biology, Faculty of Sciences, Universidad Autónoma de Madrid, 28049 Madrid, Spain; 3 Department of Immunology, Ophthalmology and Otorhinolaryngology, School of Medicine, Universidad Complutense de Madrid, 28040 Madrid, Spain; atorr01@ucm.es (A.T.-G.); melafuente@med.ucm.es (E.M.L.); 4 Instituto de Investigación Sanitaria Hospital 12 de Octubre (i+12), 28041 Madrid, Spain; 5 Department of Obstetrics and Gynecology, Hospital Universitario Quironsalud Madrid, 28223 Madrid, Spain; ricardo.sainz@quironsalud.es; 6 Instituto de Investigación Sanitaria Hospital La Princesa, 28006 Madrid, Spain

**Keywords:** exosomes, extracellular vesicles, peritoneal metastasis, colorectal cancer, adhesion molecules, ADAM17/TACE, integrin α5β1, tetraspanin CD9

## Abstract

Approximately 25% of colorectal cancer (CRC) patients develop peritoneal metastasis, a condition associated with a bleak prognosis. The CRC peritoneal dissemination cascade involves the shedding of cancer cells from the primary tumor, their transport through the peritoneal cavity, their adhesion to the peritoneal mesothelial cells (PMCs) that line all peritoneal organs, and invasion of cancer cells through this mesothelial cell barrier and underlying stroma to establish new metastatic foci. Exosomes produced by cancer cells have been shown to influence many processes related to cancer progression and metastasis. In epithelial ovarian cancer these extracellular vesicles (EVs) have been shown to favor different steps of the peritoneal dissemination cascade by changing the functional phenotype of cancer cells and PMCs. Little is currently known, however, about the roles played by exosomes in the pathogenesis and peritoneal metastasis cascade of CRC and especially about the molecules that mediate their interaction and uptake by target PMCs and tumor cells. We isolated exosomes by size−exclusion chromatography from CRC cells and performed cell-adhesion assays to immobilized exosomes in the presence of blocking antibodies against surface proteins and measured the uptake of fluorescently-labelled exosomes. We report here that the interaction between integrin α5β1 on CRC cells (and PMCs) and its ligand ADAM17 on exosomes mediated the binding and uptake of CRC-derived exosomes. Furthermore, this process was negatively regulated by the expression of tetraspanin CD9 on exosomes.

## 1. Introduction

Exosomes are a type of extracellular vesicle (EV) of endocytic origin that are released by virtually all cells in multicellular organisms and carry out important intercellular communication functions through the transfer of their biomolecular cargo, which includes lipids, proteins, nucleic acids and metabolites, between the producing and the recipient/target cells [1]. Exosomes produced by cancer cells have been shown to influence many processes related to cancer progression and metastasis, such as tumor cell proliferation and invasion, angiogenesis, tumor microenvironment promotion and remodelling, chemotherapy resistance, and immune suppression (recently reviewed in [2,3,4]).

Colorectal cancer (CRC) has a high incidence and is a major cause of cancer-related mortality worldwide, with approximately 25% of patients developing peritoneal metastasis, a condition associated with a bleak prognosis as current treatments are less effective in these patients [5]. The CRC peritoneal dissemination cascade involves sequential shedding of cancer cells from the primary colonic or rectal tumor, their transport through the peritoneal cavity following the physiological flow of peritoneal fluid, their adhesion to the peritoneal mesothelial cells (PMCs) that form a barrier lining all peritoneal organs, and the subsequent invasion of cancer cells through this mesothelial cell barrier and the underlying stroma to establish new metastatic foci in the peritoneum, omentum and bowel serosa. In addition to acting as a barrier, PMCs also play an active role in the pathogenesis of peritoneal metastasis by differentiating into cancer-associated fibroblasts (CAFs) through a process termed Mesothelial-to-Mesenchymal Transition (MMT), which fuels peritoneal metastasis and immune evasion (reviewed in [5]).

In epithelial ovarian cancer, which predominantly disseminates via peritoneal metastasis, exosomes produced by ovarian cancer cells favor different steps of the peritoneal dissemination cascade and have therefore been recognized to play crucial roles in the pathogenesis of the disease [6,7]. Of particular relevance, cancer-derived exosomes in malignant ascites interact with cancer cells promoting their survival, migration and invasion capacities, and with PMCs inducing their apoptosis, disrupting the mesothelial barrier and reprogramming them into CAFs through MMT. For CRC, however, current knowledge about the roles played by exosomes in the pathogenesis and peritoneal metastasis cascade is sparse. At the same time, the molecules that mediate the interactions between cancer-derived exosomes and their target PMCs and tumor cells mostly remain obscure.

Previously we employed the human colorectal adenocarcinoma Colo-320 cell line [8] as a useful model to study cancer-related processes such as tumorigenesis, metastasis, and tumor cell adhesion, as well as the functions and interplay between tetraspanins and cell adhesion molecules [9,10,11,12,13,14]. Colo-320 cells possess the distinctive feature of lacking endogenous expression of tetraspanin CD9 while they abundantly express integrin α5β1 but not many other members of the β1 integrin subfamily. CD9 is a widely distributed tetraspanin that associates with other transmembrane proteins, including integrins and ADAM metalloproteases in TEMs (Tetraspanin-Enriched Microdomains) (reviewed in [15]). CD9 is involved in cell adhesion, motility, sperm–egg fusion, tumorigenesis and metastasis and an inverse correlation between expression of CD9 and metastatic potential and patient survival rate has been established in many types of cancer, including CRC (reviewed in [16]).

Several reports indicate that integrin α5β1 can engage in *cis* (on the same cell) and *trans* (on different cells) interactions with the distintegrin (Dis) domain of the metalloproteinase ADAM17/TACE [14,15,17,18]. Our group showed that the tetraspanin CD9 negatively regulated integrin α5β1-mediated adhesion of cancer cells both to its canonical ligand fibronectin and to ADAM17 as a novel ligand [14,15]. Furthermore, we and others have reported that CD9 interacts directly with ADAM17 on the cell surface and regulates negatively both the sheddase and adhesive activities of this metalloproteinase [12,13,14,15,19,20,21]. Therefore, we decided to investigate whether integrin α5β1 and ADAM17 interaction and the regulatory effects of CD9 also played a role in mediating the binding of exosomes and their uptake by cancer or mesothelial recipient cells, which could bear relevance during the peritoneal dissemination of colorectal carcinomas.

We report here that the interaction between integrin α5β1 on colorectal carcinoma cells and on PMCs and its ligand ADAM17 on exosomes mediates the binding and uptake of cancer-derived exosomes. Furthermore, this process was negatively regulated by the expression of tetraspanin CD9 on the surface of exosomes.

## 2. Results

### 2.1. Characterization of EVs Derived from Colo-320, Colo-320/CD9 and Colo-320/ADAM17-KO Human Adenocarcinoma Cells

**Figure 1:**
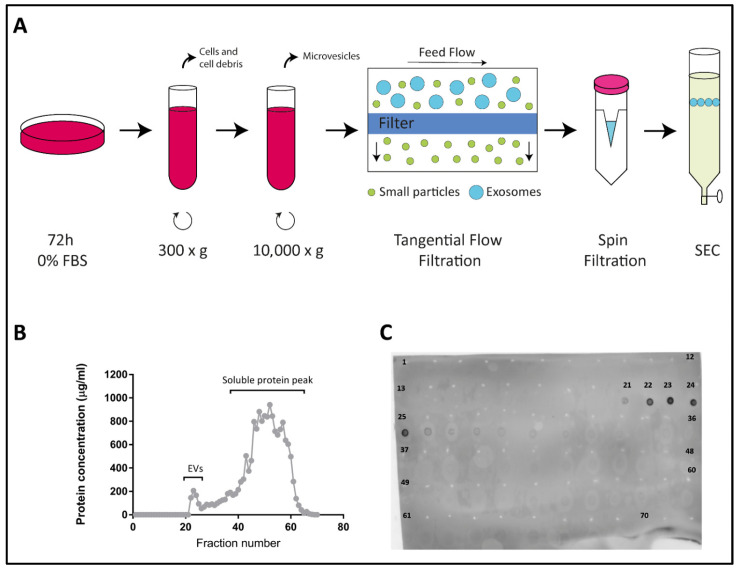
Isolation of exosomes from Colo-320 cells. (**A**) Experimental procedure for isolation of exosomes from conditioned medium of Colo-320 cells. Cells were cultured in serum-free conditions for 72 h before collection of the medium, which was then sequentially centrifuged at 300× *g* to remove cells and large debris and at 10,000× *g* to remove larger EVs such as microvesicles. Medium was then concentrated by tangential flow filtration and spin filtration to a final 1.0 mL volume. Exosomes were finally isolated by size exclusion chromatography (SEC). (**B**) A representative SEC elution profile showing the protein concentration of the 70 fractions (0.5 mL each) typically collected. (**C**) Dot blot showing the fractions containing exosomes, based on the presence of the exosomal marker CD81.

We have previously described the generation of two stable lines derived from parental human colorectal adenocarcinoma Colo-320 cells: Colo-320/CD9, expressing high levels of cell surface CD9 after stable transfection; and Colo-320/ADAM17-KO, lacking expression of the metalloproteinase ADAM17/TACE after specific CRISPR/Cas9 knock-out of this enzyme [14] (see also supplementary material). EVs were isolated from FBS-free conditioned media of Colo-320, Colo-320/CD9 and Colo-320/ADAM17-KO cells, by a protocol that included two sequential centrifugations, first at 300× *g* to discard whole cells and large cellular debris, and at 10,000× *g* to discard the pellet containing particulate material and larger extracellular vesicles such as microvesicles. The resultant supernatants were [20] concentrated by tangential flow and spin filtrations and EVs purified by size−exclusion chromatography (SEC) fractionation in a 30 mL Sepharose Fast-Flow 4 column (Figure 1A and described in detail in Materials and Methods). Typically, a total of 70 fractions (0.5 mL each) were collected from each sample and their protein content was quantified using the Micro BCA Protein Assay Kit. Figure 1B shows a representative graph of the protein content of all collected fractions with two clearly distinguishable peaks: a small first peak, which corresponded to the elution of EVs, typically comprising fractions 21–25, and a much larger second peak (fractions 26–70) corresponding to the elution of free soluble protein. The exosomal content of the fractions in the first peak was confirmed by dot blot analysis using an antibody against the tetraspanin CD81, a classical EV marker (Figure 1C) [22,23].

**Figure 2:**
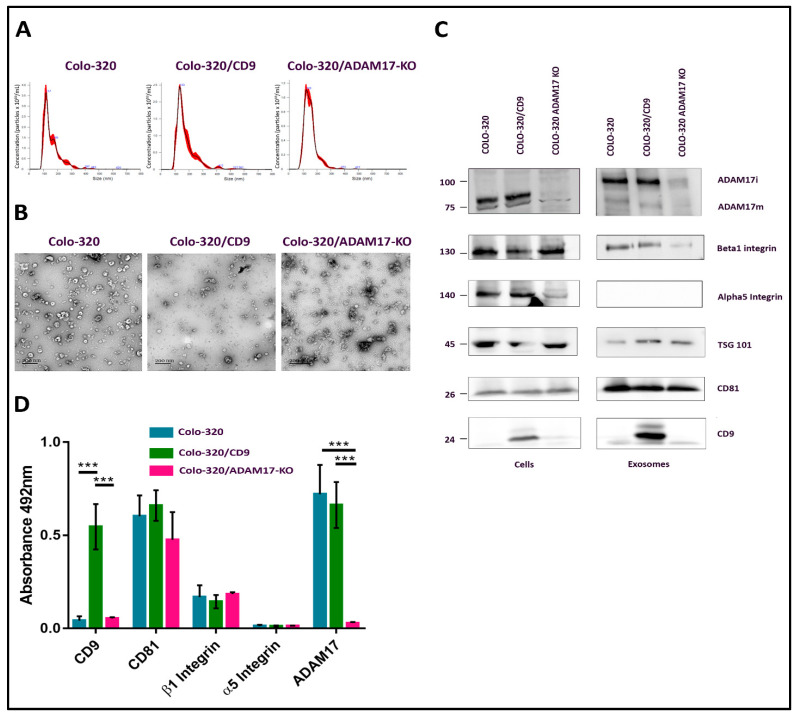
Characterization of exosomes produced by Colo-320 cells. (**A**) NTA analysis of the number and size of particles (exosomes) produced by Colo-320, Colo-320/CD9 and Colo-320/ADAM17-KO. (**B**) Transmission electron microscopy of exosomes produced by Colo-320, Colo-320/CD9 and Colo-320/ADAM17-KO. Scale bars= 200 nm. (**C**) Western blot showing the expression of ADAM17, CD9, CD81, TSG101, integrin β1 and integrin α5 in Colo-320, Colo-320/CD9 and Colo-320/ADAM17-KO cells and in their respective exosomes. Blots are representative of three different experiments. (ADAM17i: the immature form of the enzyme that contains the prodomain; ADAM17m: the mature form without the prodomain). (**D**) Detection of CD9, CD81, integrin β, integrin α5 and ADAM17 by enzyme-linked immunosorbent assay (ELISA) on immobilized exosomes produced by Colo-320, Colo-320/CD9 and Colo-320/ADAM17-KO cells. Data correspond to the mean ± SEM of three different experiments, each performed in duplicates. Statistical analysis performed was two-way ANOVA *** *p* < 0.001.

The CD81-positive fractions of the first peak (fractions 21–25) were pooled and the size distribution and concentration of EVs in the resulting samples were determined by Nano Tracking Analysis (NTA) (Figure 2A). Typically, EV concentration in pooled samples was around 1–4 × 10^10^ particles/mL, and the diameter of the particles fell within the range of 70–150 nm, as expected for exosomes. The round cup-like morphology of these particles, as visualized by transmission electron microscopy after negative staining, also corresponded to exosomes (Figure 2B). The presence of CD81, CD9, TSG101, α5 integrin, β1 integrin and ADAM17 in exosome samples and in the producing cells was detected by western blotting, using specific antibodies (Figure 2C). In addition, the content of CD81, CD9, α5 integrin, β1 integrin and ADAM17 in these exosome samples was also quantified after their immobilization on plastic wells (5 μg of total EV protein/well) by an indirect ELISA assay using specific primary antibodies to these proteins and HRP-conjugated secondary antibody (Figure 2D). CD81 and TSG101, which are considered *bona fide* exosomal markers [21], were detected abundantly in the exosomes derived from the three cell lines. As expected, CD9 was detected only in exosomes derived from the CD9-positive Colo-320/CD9 cells, but not in exosomes derived from the CD9-negative Colo-320 or Colo-320/ADAM17-KO cells. Surprisingly, while the integrin β1 subunit was readily detected both in cell and EV lysates by western blot (Figure 2C), on the plasma membrane of the three cell lines (assessed by flow cytometry, Supplementary Figure S1) and in the exosomes derived from them (assessed by western blot and ELISA, Figure 2C,D), the integrin α5 subunit, despite being expressed abundantly in cell lysates (Figure 2C) and on the surface of the three cell lines (Supplementary Figure S1) could not be detected (either by WB or by ELISA) in the exosomes derived from any of them (Figure 2C,D). These results could indicate that in these cells the integrin α5 was actively excluded from exosomes by a process of selective protein sorting.

### 2.2. Fibronectin Is Not the Ligand of Integrin α5β1 That Mediates Interactions of Exosomes with Colo-320 Cancer Cells

**Figure 3:**
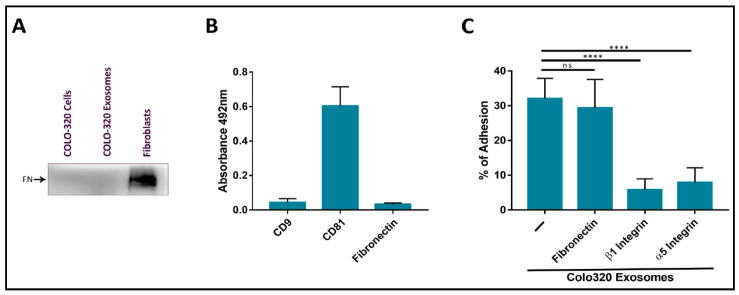
The adhesion of CRC Colo-320 cells to exosomes was specifically mediated by integrin α5β1 but not by fibronectin. (**A**) Western Blot of Colo-320 cells and their exosomes shows lack of expression of fibronectin. As a positive control, abundant fibronectin was detected in a fibroblasts lysate. Blots are representative of three different experiments. (**B**) Detection by enzyme-linked immunosorbent assay (ELISA) of CD9, CD81 and fibronectin on immobilized exosomes produced by Colo-320 cells. (**C**) Cell adhesion of Colo-320 cells to immobilized exosomes produced by these same cells was blocked by mAbs specific for integrin β1 or integrin α5, but not for fibronectin. The percentage of cells that remained adhered is indicated as mean ± SEM of four experiments, performed in duplicates. Statistical analysis performed was one-way ANOVA. **** *p* < 0.0001, ns: not statistically significant.

Several groups have reported that exosomes produced by different cell types, including cancer cells, are coated with the extracellular protein fibronectin, and that this coat plays important roles in the interaction between these EVs and recipient/target cells and in directing cell movement through the tissues [24,25,26,27,28]. The origin of the fibronectin found on exosomes can be endogenous from biosynthesis in the producing cells or exogenous from the bovine fibronectin present in FBS added to culture medium. In order to exclude exogenous fibronectin, we routinely deprived Colo-320 cancer cells of FBS for 72 h before exosome isolation. Following this protocol, we could not detect the presence of fibronectin on the surface of Colo-320 cancer cells by flow cytometry (Supplementary Figure S1) or in cell lysates by western blot (Figure 3A), which would indicate that these cells do not biosynthesize this protein. As a positive control, we detected abundant fibronectin on the surface of human fibroblasts cultured under the same conditions (72 h of FBS-deprivation) both by flow cytometry (Supplementary Figure S2A) and in cell lysates by western blot (Figure 3A). Therefore, as expected from the lack of fibronectin biosynthesis in these cells, this ECM protein could not be detected on the exosomes released by Colo-320 cells by either western blot (Figure 3A) or by ELISA (Figure 3B).

We thereafter quantified the adhesion of Colo-320 cells to immobilized exosomes produced by the same cells. These adhesion assays had been previously employed by other groups to study the interactions of exosomes with recipient/target cells and led to the identification of some of the receptor and ligand molecules mediating such interactions [25]. In these adhesion assays, around 35–40% of PMA-stimulated Colo-320 cells efficiently adhered to immobilized exosomes. Cell adhesion was almost completely abrogated by using blocking mAbs against the β1 (Lia1/2) or α5 (P1D6) chains of the fibronectin receptor integrin α5β1 (Figure 3C), indicating that this adhesion molecule played a major role in these cell−exosome interactions. In contrast, a blocking anti human fibronectin mAb (HFN7.1), which completely knocked out the adhesion of Colo-320 cells to immobilized fibronectin (Supplementary Figure S2B), did not significantly reduce cell adhesion to immobilized exosomes (Figure 3C). These results concur with the observed lack of fibronectin content both in Colo-320 cells and in the exosomes derived from them and demonstrate that interactions of exosomes with Colo-320 cells were mediated by integrin α5β1 but not through the binding to its canonical ligand fibronectin.

### 2.3. Expression of CD9 Reduces Interactions between Cancer Cells and Exosomes Mediated by Cellular Integrin α_5_β_1_ and Exosomal ADAM17

**Figure 4:**
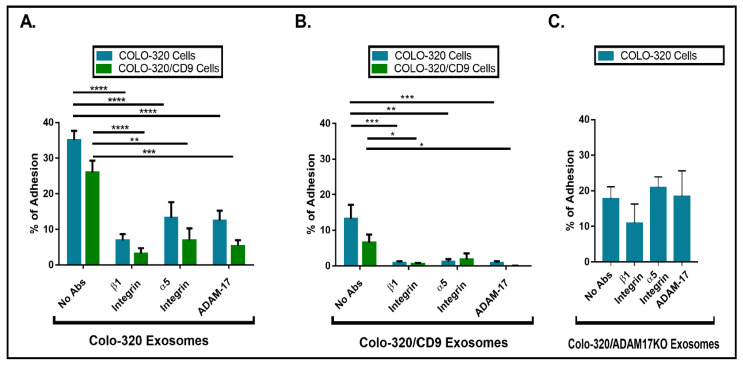
The presence of CD9 reduced cell adhesion to immobilized exosomes that was mediated by cellular integrin α5β1 and exosomal ADAM17. Cell adhesion of Colo-320 or Colo-320/CD9 to exosomes produced by Colo-320 cells (**A**), Colo-320/CD9 cells (**B**), and Colo-320/ADAM17-KO cells (**C**). Cells were stimulated with phorbol ester PMA (200 ng/mL) for 1 h, loaded with the fluorescent probe BCECF-AM and then allowed to adhere to immobilized exosomes (5 μg of exosomal protein per well) for 60 min at 37 °C under physiological conditions of Ca^2+^/Mg^2+^ (0.5 mM/1 mM, respectively). The percentage of cells that remained adhered is indicated as mean ± SEM of four experiments, performed in duplicates. Statistical analysis performed was two-way ANOVA. * *p* < 0.05, ** *p* < 0.01, *** *p* < 0.001, **** *p* < 0.0001.

The Disintegrin (Dis) domain of metalloproteinase ADAM17 has been reported by several groups, including ours, as a novel ligand for integrin α5β1 [14,17,18,29,30]. Therefore, we investigated whether Dis-ADAM17 could be the integrin α5β1 ligand mediating the interactions of exosomes with Colo-320 cancer cells. As shown in Figure 4A, a blocking antibody (mAb 2A10) specific for the Dis domain of ADAM17 [14] significantly inhibited the adhesion of Colo-320 cells to immobilized exosomes. This effect was almost identical to the inhibition caused by blocking the integrin α5β1 with the P1D6 mAb. Taking into account that the only adhesion molecule described to interact in *trans* with ADAM17 was the integrin α5β1, and also that the α5 chain of this integrin was abundantly expressed on Colo-320 cells (Supplementary Figure S1) but not on exosomes (Figure 2C,D), these results strongly indicate that the Dis domain of exosomal ADAM17 was the ligand being recognized by cellular integrin α5β1 during interactions of exosomes with Colo-320 cancer cells.

CD9 negatively regulates integrin α5β1-mediated cell adhesion to both its canonical ligand fibronectin and to the Dis domain of ADAM17 (Dis-ADAM17) [14]. Therefore, we decided to assess whether CD9 could also regulate the *trans* interaction of exosomal Dis-ADAM17 with cellular integrin α5β1. Firstly, we compared the capacity of exosomes either lacking (produced by parental Colo-320 cells) or expressing CD9 (produced by Colo-320/CD9 cells) to interact with Colo-320 and Colo-320/CD9 cells by allowing these cells to adhere to exosomes immobilized on plastic wells. The results of these assays (Figure 4A,B) show that expression of CD9 on the cell surface resulted in a moderate but consistent reduction in their capacity to adhere to exosomes. More dramatic was the reduction of adhesion of Colo-320 and Colo-320/CD9 cells to exosomes when these EVs expressed CD9 on their surface, as cell adhesion dropped from 35% (Figure 4A) to 13% (Figure 4B). In all cases, cell adhesion was significantly inhibited by blocking mAbs specific for the β1 and α5 integrin subunits and for the Dis domain of ADAM17. These data establish that expression of CD9 on exosomes negatively regulates the capacity of Dis-ADAM17 to act as a ligand of integrin α5β1, and are in full agreement with the previously described effects of CD9 expression on ADAM17 on the surface of Colo-320 cells [14].

The fact that expression of integrin α5β1 was high on the cell surface but minimal on the surface of exosomes pointed to a specific *trans* configuration, involving cellular integrin α5β1 and exosomal Dis-ADAM17 as molecules mediating these interactions of EVs with Colo-320 cells. To further prove this point, we assessed the adhesion of Colo-320 cells to immobilized exosomes lacking ADAM17 expression (isolated from Colo-320/ADAM17-KO cells). As shown in Figure 4C, adhesion of Colo-320 cells to ADAM17-KO exosomes (Colo-320/ADAM17-KO exosomes) was noticeably reduced compared to the adhesion to exosomes expressing ADAM17 (Colo-320 exosomes) (Figure 4A), demonstrating the crucial involvement of exosomal ADAM17 in these cell−exosome interactions. Importantly, a lack of ADAM17 expression on exosomes rendered these cell−exosome interactions completely resistant to the blocking effects of anti-α5 or anti-Dis-ADAM17 mAbs, confirming the involvement of ADAM17 on the exosomal side in such interactions and indicating that when exosomal ADAM17 was absent other adhesion molecule pairs may have taken over in mediating cell−exosome interactions.

### 2.4. Uptake of Exosomes by Colo-320 Cancer Cells Depends on Exosomal ADAM17 and Is Inhibited by CD9

**Figure 5:**
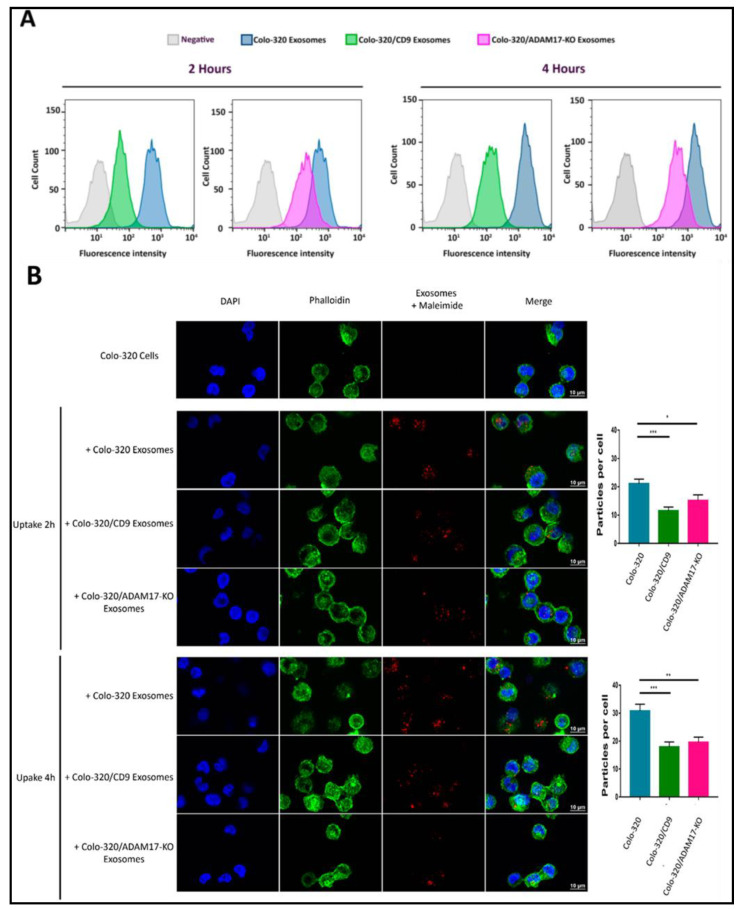
Expression of exosomal CD9 and knocking-out ADAM17 expression in exosomes reduced the uptake of exosomes by Colo-320 recipient cells. (**A**) Flow cytometry quantitation of the uptake by Colo-320 recipient cells of fluorescently labelled exosomes produced by Colo-320, Colo-320/CD9 and Colo-320/ADAM17-KO cells, after 2 and 4 h or cell−exosome co-incubation. (**B**) Confocal microscopy images showing the uptake by Colo-320 recipient cells of the different exosomes as in (**A**). The bar graphs show the quantification of exosomal uptake by counting the particles internalized by at least 80 cells for each condition in three separate experiments. Statistical analysis performed was one-way ANOVA. * *p* < 0.05, ** *p* < 0.01 *** *p* < 0.001.

Our results showing that binding of cellular integrin α5β1 to its exosomal ligand Dis-ADAM17 mediated the initial interaction of exosomes with recipient cancer cells and that this process was negatively regulated by CD9 prompted us to investigate if these molecules also had an effect in the subsequent exosome uptake by these cells. For this purpose, we first employed a flow cytometry assay to quantitatively assess the uptake by Colo-320 cells of fluorescently labeled exosomes [31]. Figure 5A shows that exosome uptake was markedly reduced after ADAM17 knock-out (Colo-320/ADAM17-KO exosomes) though not as pronounced as the reduction observed upon CD9 expression (Colo-320/CD9 exosomes). This reduction in the uptake was evident both after 2 h and 4 h of co-incubation of exosomes and cells. We also confirmed, using confocal microscopy, that exosomes derived from cells with deletion of ADAM17 or neo-expression of CD9 greatly reduced their uptake by recipient Colo-320 cancer cells (Figure 5B). Although the limit of resolution of confocal microscopes is above the exosomal size, the fluorescent signal detected corresponded to aggregates of exosomes that accumulate in endosomal compartments after their uptake, as evidenced by their colocalization with the tetraspanin CD63 [32].

### 2.5. Interactions between Colorectal Carcinoma Cells-Derived Exosomes and Peritoneal Mesothelial Cells Are Mediated by ADAM17 and Integrin α5β1 and Regulated by CD9 Expression

**Figure 6:**
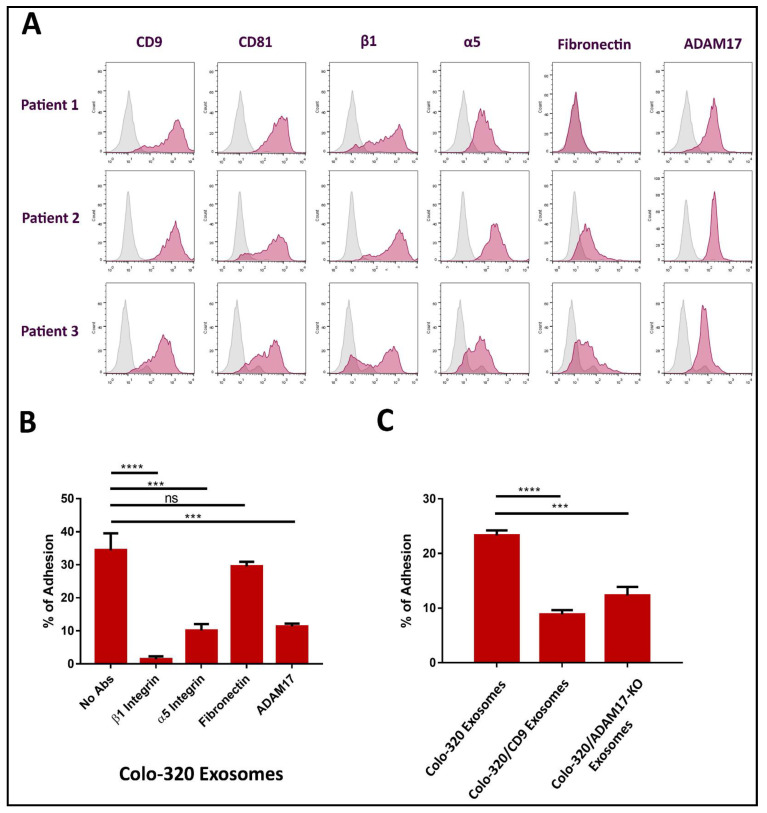
The presence of exosomal CD9 and the absence of exosomal ADAM17 reduced the adhesion of primary mesothelial cells to exosomes produced by CRC Colo-320 cells. (**A**) Flow cytometry detection of CD9, CD81, integrin β1, integrin α5, ADAM17, and fibronectin on the surface of primary mesothelial cells from three different donors. (**B**) Adhesion of primary mesothelial cells (from the three patients in (**A**)) to immobilized exosomes produced by Colo-320 cells in presence of blocking mAbs specific for β1 integrin, α5 integrin, fibronectin and the Dis domain of ADAM17. (**C**) Adhesion of primary mesothelial cells (from the three patients in (**A**)) to immobilized exosomes produced by Colo-320, Colo-320/CD9 and Colo-320/ADAM17-KO cells. The percentage of cells that remained adhered is indicated as mean ± SEM of three experiments (one for each patient), performed in duplicates. Statistical analysis performed was one-way ANOVA. *** *p* < 0.001, **** *p* < 0.0001, ns: not statistically significant.

Colorectal carcinomas frequently use dissemination and implantation in the peritoneal cavity. This process is termed peritoneal carcinomatosis and is related to an advanced stage of colorectal cancer [5,33]. To investigate if ADAM17 also plays a role in the interaction between colorectal cancer exosomes and PMCs, we obtained primary human PMCs from omentum samples of patients undergoing abdominal surgery. As shown in Figure 6A, we found that CD9, CD81, integrin α5β1 and ADAM17 molecules were highly expressed on the cell surface of PMCs. In contrast, fibronectin expression showed a great variation among patients, ranging from completely undetectable to being abundantly expressed. In accordance with our previous results, adhesion of these PMCs to immobilized exosomes from Colo-320 cells was significantly diminished by blocking mAbs specific for β1 (Lia1/2) or α5 (P1D6) integrin subunits, and for the Dis domain of ADAM17 (2A10), but remained unaffected after blocking fibronectin with mAb HFN7.1 (Figure 6B). These results demonstrate that interactions between colorectal cancer-derived exosomes and primary PMCs were also mediated by the Dis domain of ADAM17 and integrin α5β1, but not by fibronectin. Similarly, adhesion of PMCs to immobilized exosomes was significantly reduced when exosomes expressed CD9 or were deficient in ADAM17 (Figure 6C).

## 3. Discussion

Exosomes produced by tumor cells play pivotal roles in the pathogenesis of ovarian cancer by promoting the peritoneal dissemination cascade through their interactions with cancer and peritoneal mesothelial cells (PMCs) [6,7]. Whether exosomes also play similar roles in the peritoneal metastasis of CRC has not been thoroughly addressed [34], although a recent study points to shared mechanisms in the peritoneal dissemination process in both types of cancer [35]. Likewise, the molecules that mediate the interactions and the uptake of cancer exosomes by recipient/target cells remain largely unknown, though adhesion molecules such as integrins and their ligands have been proposed to be involved in these processes [36,37]. In fact, distinct exosomal integrins have been shown to determine the uptake of tumor exosomes by specific target cells, thus dictating the metastatic tropism of different types of cancer [38].

To gain insight about the molecules involved in the interactions and uptake of exosomes (produced by CRC cells) by target PMCs and cancer cells, in this study we employed the human adenocarcinoma Colo-320 cell line. These cells represent a very useful model to study the regulation exerted by the tetraspanin CD9 on cell adhesion molecules of the integrin, immunoglobulin and ADAM families, which are relevant to cancer-related processes such as tumorigenesis and metastasis, because they lack endogenous expression of CD9 but abundantly express the integrin α5β1 [9,10,11,12,13,14]. From these parental Colo-320 cells we previously generated two stable cell lines, Colo-320/CD9 and Colo-320/ADAM17-KO, which allowed us to study here the specific involvement of CD9 and ADAM17 in the interactions and uptake of tumor exosomes by recipient cells [14]. Through the use of these three CRC Colo-320 cell lines, our results have established: (i) that the interaction between integrin α5β1 −expressed on CRC cells or on PMCs− and its novel ligand Dis-ADAM17 −exposed on exosomes− mediates the binding and uptake of exosomes produced by CRC Colo-320 cells; and (ii) that this process is negatively regulated by the expression of tetraspanin CD9 on the surface of exosomes.

Several reports have shown that fibronectin coats the surface of cancer-derived exosomes, being recognized by specific cellular receptors, such as integrin α5β1 or heparan sulphate proteoglycans, thus mediating exosome-cell interactions involved in cell invasion and movement through the tissues [24,25,26,27,28]. In contrast to these reports, in the present study we have ruled out a role for exosomal fibronectin in mediating cell−exosome interactions and exosomal uptake, as the tumor Colo-320 cells (and the cell lines derived from them) appeared not to be able to biosynthesize fibronectin and because the exosomes produced by these cells did not bind exogenous fibronectin under the FBS-deprivation conditions employed throughout our study. In this regard, we have established here that the ligand being recognized by cellular integrin α5β1 on Colo-320-produced exosomes during cell−exosome interactions and exosome uptake is the Dis domain of the metalloproteinase ADAM17/TACE, and not fibronectin. Thus, upon elimination of ADAM17 expression on exosomes produced by Colo-320/ADAM17-KO cells, cell−exosome interactions were diminished and no longer were mediated by cellular integrin α5β1, indicating that other pairs of adhesion receptor-ligand molecules could take over in the absence of ADAM17.

**Figure 7:**
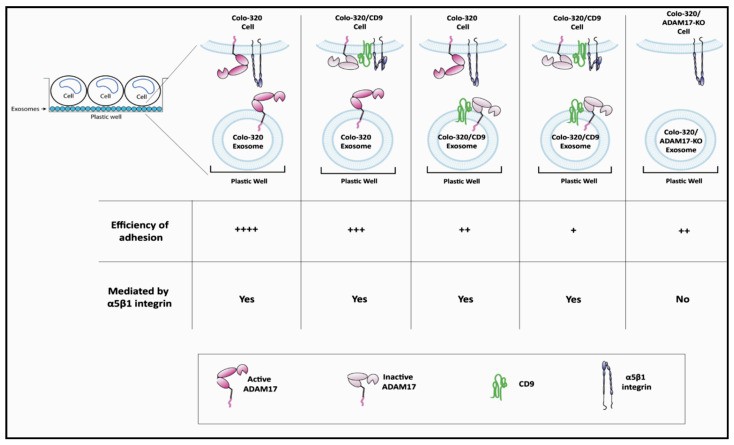
Schematic representation of the involvement of ADAM17, CD9 and integrin α5β1 in interactions between exosomes and cells. The interaction of exosomes with cells was mediated by integrin α5β1 in cells and ADAM17 in exosomes and was regulated by CD9. Cells adhered more efficiently to exosomes lacking CD9. Knocking-out ADAM17 expression in exosomes reduced cell−exosome interactions, which were no longer mediated by cellular integrin α5β1. (Efficiency of adhesion is comparatively assigned: **++++** denotes highly efficient adhesion; **+++** denotes efficient adhesion; **++** denotes less efficient adhesion; **+** denotes least efficient adhesion).

Regarding the negative regulation exerted by the expression of exosomal CD9 on the capacity of ADAM17 to support cell−exosome interactions and exosome uptake, we and others have previously reported that this tetraspanin is engaged in direct *cis* interactions with ADAM17 on the cell surface [12,19,20,21], and through this lateral association CD9 inhibits both the sheddase and adhesive activities of ADAM17 [12,14]. The mechanism, by which expression of CD9 regulates negatively the capacity of ADAM17 to support cell adhesion, seems to be related to changes induced by CD9 in the organization and size of discrete clusters of this metalloproteinase, which impinge on the overall avidity of cell adhesion [14]. Our results in the present study show that similar regulatory mechanisms were exerted by CD9 on the adhesion-supporting capacity of ADAM17 when this tetraspanin was expressed on the surface of cancer-derived exosomes (Figure 7).

One limitation of our study was that only exosomes from CRC Colo-320 cells (and derived cell lines: Colo-320/CD9 and Colo-320/ADAM17-KO) were used because this experimental system allowed a detailed analysis of the relevance of CD9, ADAM17, and integrin α5β1 molecules. It would be important to validate the results obtained here by using tumoral exosomes produced by other CRC cell lines or even by cell lines of other cancer types, such as ovarian or gastric cancer, that also disseminate very frequently via peritoneal metastasis.

The expression of CD9 is diminished in gastric cancer and has been proposed to be inversely associated with lymph node metastasis and with increased recurrence risk [39,40,41]. These findings would concur with our results showing that the lack of CD9 expression on exosomes produced by CRC cells enhanced their interaction and uptake by recipient cells.

In sum, our data unveil that the interaction of cellular integrin α5β1 with ADAM17 exposed on the surface of exosomes is negatively regulated by CD9 and plays a role in EV adhesion and uptake by CRC cancer cells and PMCs, suggesting that these molecules could be relevant therapeutic targets to inhibit CRC peritoneal carcinomatosis. In this regard, integrin α5β1 has been established as a target molecule for therapy in epithelial ovarian and primary peritoneal cancer and small molecule inhibitors and function-blocking monoclonal antibodies for this integrin are being investigated in clinical trials (reviewed in [42]). Similarly, CD9-targeting antibodies have been investigated as anti-cancer agents in different types of tumors (reviewed in [16]) and CD9 has been proposed as a therapeutic target in gastrointestinal cancer [41]. Finally, the implication of ADAM17 in different types of cancer is beginning to unravel and strategies to therapeutically target this metalloproteinase are being developed (reviewed in [43]).

## 4. Materials and Methods

### 4.1. Cells and Antibodies

Colo-320 (colorectal adenocarcinoma) human cell lines were cultured in RPMI-1640 supplemented with 10% heat-inactivated FBS, 2 mM glutamine, 50 μg/mL streptomycin and 50 U/mL penicillin. The human fibroblast cell line (GM08402, Coriell Institute Cell Repositories) was cultured in DMEM supplemented with 10% heat-inactivated FBS, 2 mM glutamine, 50 μg/mL streptomycin, and 50 U/mL penicillin. Generation of Colo-320/CD9 (stably transfected with CD9 cDNA) and Colo-320/ADAM17-KO (ADAM17 gene knocked out by CRISPR/Cas-9) cell lines from the parental Colo-320 cells has been previously reported [11,14] and is also included in the supplementary material.

Human peritoneal mesothelial cells were obtained from omentum samples of patients undergoing elective surgery, as previously described [44,45]. Mesothelial cells were cultured in Earle’s M199 medium supplemented with 20% fetal bovine serum, 50 U/mL penicillin, 50 μg/mL streptomycin, and 2% Biogro-2 (Biological Industries, Beit HaEmek, Israel).

Generation of monoclonal antibodies 2A10 (specific for the Disintegrin domain of human ADAM17) [12,14], A300E mAb (specific for the Membrane-Proximal domain of human ADAM17) [46], P1D6 (anti-α5 integrin) [47], TS2/16 (anti-β1 integrin), Lia1/2 (anti-β1 integrin) [48,49], PAINS-10 (anti-CD9) [9], 5A6 (anti-CD81) [50], and HFN 7.1 (anti-Fibronectin) [51] has been reported elsewhere. mAbs were purified by protein A- or protein G-affinity chromatography, according to their isotype.

### 4.2. Flow Cytometry

Cells were incubated with the corresponding primary antibodies for 30 min at 4 °C, washed three times in RPMI-1640 and incubated with the secondary polyclonal antibody Alexa Fluor^®^647-conjugated Goat anti-mouse IgG (Thermo Fisher, Madrid, Spain) for 30 min at 4 °C. Cells were washed three times and then fixed in 2% formaldehyde in PBS. Fluorescence was measured using a FACScan™ flow cytometer (Beckton-Dickinson, Madrid, Spain).

### 4.3. Extracellular Vesicles Isolation

Extracellular vesicles (EVs) were isolated from 200 mL of culture medium of Colo320 cell lines (seeded at 1 × 10^6^ cells/mL), deprived of FBS for 72 h. Briefly, the medium was centrifuged at 300× *g* for 5 min to eliminate cells and large debris, and the supernatant was subjected to centrifugation at 10,000× *g* for 15 min to eliminate larger EVs (microvesicles). The supernatant was then concentrated by tangential flow filtration using a Vivaflow 50 R membrane (Sartorius, Goettingen, Germany) to a volume of 15 mL, and further concentrated using a 100 kDa Amicon Ultra-15 Centrifugal filter (Merck, Darmstadt, Germany) to a volume of 1.0 mL, which was finally applied on top of a 30 mL column of Sepharose 4 Fast-Flow resin for size−exclusion chromatography (SEC) and eluted with filtered PBS. Typically, a total of 70 fractions (0.5 mL each) were collected from each sample.

### 4.4. Dot Blot

Detection of the proteins of all fractions collected from SEC were assesed by Dot-blot. From each fraction, 2 μL was spotted on a nitrocellulose membrane (Pall Life Science, Portsmouth, UK). Membranes were blocked with 3% BSA and incubated with primary mAb 5A6 (anti-CD81) (1 µg/mL), followed by three washes with 0.1% Tween20-TBS and incubation with secondary polyclonal antibody HRP-conjugated goat anti-mouse IgG (Sigma-Aldrich Merck, Madrid, Spain) and incubated with ECL Plus western blotting Detection System (GE Healthcare, Madrid, Spain) for ECL-chemiluminescence detection in an ImageQuant LAS4000 biomolecular imager (GE Healthcare).

### 4.5. Nano Tracking Analysis (NTA)

The size and the concentration of EVs were assessed by Nanoparticle Tracking Analysis (NTA) in a Nanosight NS500 (Malvern Instruments Ltd., Malvern, UK), after diluting 1/100 in filtered PBS the samples eluted from SEC. A total of three videos of 1 min were acquired for each sample.

### 4.6. Transmission Electron Microscopy

For Transmission Electron Microscopy, exosomes were negatively stained with 2% uranyl acetate in double-distilled water for 45 s before visualization. Exosomes were visualized in a Jeol JEM-1010 transmission electron microscope and the images were acquired with a 4KI 4K CMOS camera, F416 of TVIPS.

### 4.7. Enzyme-Linked Immunosorbent Assay (ELISA)

For ELISA assays, 96-well flat-bottom plates were pre-coated overnight at 4 °C with exosomes (5 μg of exosomal protein per well) and then blocked for 1 h with 1% BSA in PBS. The wells were incubated with primary mAbs PAINS-10 (anti-CD9), 5A6 (anti-CD81), TS2/16 (anti-β1), P1D6 (anti-α5), HFN 7.1 (anti-fibronectin) or 2A10 (anti-Dis domain of ADAM17) at 20 μg/mL for 3 h. The plates were then washed three times with 0.05% Tween20-PBS and 50 µL of a 1/500 dilution in PBS of commercial secondary polyclonal antibody HRP-conjugated goat anti-mouse IgG (Sigma-Aldrich Merck) was added to each well and incubated for 1 h at RT. After washing with 0.05 Tween 20 in PBS, 100 μL of developing solution (40 mg of o-Phenylenediamine dihydrochloride substrate -OPD- in 10 mL of 0.2 M NaHPO4, 0.1 M citric acid, and 40 μL 3% H_2_O_2_). The reaction was stopped with 100 μL of 0.25 mM HCl and absorbance at 492 nm was determined with a Tecan GeNIOS plate reader.

### 4.8. Western Blot

Cells were lysed for 30 min at 4 °C in 1% Triton-X100 TBS with Protease Inhibitor Cocktail (Sigma-Aldrich Merck, Madrid, Spain), centrifuged at 10,000× *g* for 5 min, and protein concentration of the cell lysates (supernatants) was quantified using the DC Protein Assay Kit (Bio-Rad Laboratories, Alcobendas, Spain). Cell lysates were mixed with Laemmli buffer and heated at 100 °C for 7 min.

Protein concentration of the EVs samples in fractions collected from SEC was determined according to the micro-BCA Assay Kit protocol instructions (Thermo-Fisher Scientific, Madrid, Spain) and the absorbance was determined in a plate reader (Tecan GeNIOS) at 540 nm. Then, EVs were lysed with Laemmli buffer and heated for 7 min at 100 °C. Lysates of cells and EVs were resolved in 6%, 8% or 10% SDS-PAGE gels and transferred to nitrocellulose membranes (Bio-Rad Laboratories, Spain). Membranes were blocked with 3% BSA, incubated with the appropriate primary antibodies, washed three times in 0.1% Tween20-TBS, incubated with secondary polyclonal antibody HRP-conjugated goat anti-mouse IgG (Sigma-Aldrich Merck) and finally incubated with ECL Plus western blotting Detection System (GE Healthcare, Madrid, Spain) prior to ECL-chemiluminescence detection in an ImageQuant LAS4000 biomolecular imager (GE Healthcare, Madrid, Spain). For detection of ADAM17 in exosomes, the ECL substrate SuperSignal West Femto Maximum Sensitivity Substrate (Thermo Scientific) was used prior to ECL-chemiluminescence detection.

### 4.9. Cell Adhesion Assays

Cell adhesion assays to plastic wells coated with exosomes produced by Colo-320, Colo-320/CD9 and Colo-320/ADAM17-KO were performed essentially as described previously for cell adhesion assays to immobilized recombinant proteins [13,14]. Exosomes (5 μg of exosomal protein per well) were immobilized in 96-well flat-bottom plates O/N at 4 °C. Cells were stimulated with phorbol ester PMA (200 ng/mL) for 1 h and loaded with the fluorescent probe BCECF-AM for 20 min at 37 °C in PBS. Cells were washed and incubated with 10% FBS-RPMI for 10 min at 37 °C and then resuspended in adhesion buffer (20 mM Hepes, 149 mM NaCl, 2 mg/mL glucose, 1 mM MgCl_2_ and 0.5 mM CaCl_2_). A total of 1.5 × 10^5^ cells in 100 µL of adhesion buffer were added to each well in the presence of the appropriate (20 μg/mL) anti-ADAM17 (2A10), anti-β1 (Lia1/2), anti-α5 (PID6) or anti-fibronectin (HFN7.1) mAbs and allowed to adhere for 40–60 min at 37 °C. The wells were gently washed (3–5 washes) and the percentage of adherent cells in each well was calculated by determining their fluorescence relative to the 100% input fluorescence (before removing the non-adherent cells) in a microplate reader (TecanGENios).

### 4.10. Uptake of Exosomes

Exosomes (100 μg/100 µL) were incubated with AlexaFluor 633-C_5_-maleimide (0.05 mM) (Thermo Fisher Scientific) O/N at 4 °C. To remove the excess of maleimide, exosomes were centrifuged in exosome spin columns with a 3000 MW cut-off (Invitrogen, Madrid, Spain), according to the manufacturer’s instructions. Then, 8 μL of eluted labeled exosomes were incubated with 1.5 × 10^5^ Colo-320 cells in a final volume of 150 µL for 2 or 4 h. For flow cytometry analysis, cells were washed and treated with 0.25–0.02% trypsin-EDTA solution for 5 min (to remove exosomes bound to the cell surface but not yet taken up by the cells) and then the fluorescence corresponding to internalized exosomes was measured in a FACScan™ flow cytometer (Beckton-Dickinson). For confocal microscopy, cells were placed on glass coverslips coated with poly-L-lysine for 45 min at 37 °C, fixed with 2% formaldehyde in PBS for 8 min, and blocked in 3% BSA-TBS for 30 min before incubation with Alexa-Fluor-488 Phalloidin (Thermo Fisher Scientific) for 30 min. Cells were washed three times to remove the excess of fluorescent Phalloidin, and then mounted on microscope slides with Fluoromount™/DAPI (Sigma-Aldrich). Images were acquired in an LSM710 vertical confocal microscope (Zeiss, Oberkochen, Germany). Fiji/Image-J software was used for analysis of fluorescent dots in microscopic images.

### 4.11. Statistical Analyses

As indicated in the individual figure legends, different statistical analyses of data were performed using the GraphPad PRISM (version 7.0) software. These analyses included the one-way ANOVA coupled with Dunnet’s multiple comparison tests, and the two-way ANOVA. The different levels of *p*-value considered for statistical significance are indicated by asterisks in the individual figure legends (* *p* < 0.05, ** *p* < 0.01, *** *p* < 0.001, **** *p* < 0.0001).

## Supplementary Materials

The following are available online at https://www.mdpi.com/article/10.3390/ijms22189938/s1.
