# Supplementary material for: Cellular Integrin α5β1 and Exosomal ADAM17 Mediate the Binding and Uptake of Exosomes Produced by Colorectal Carcinoma Cells
Source: Int J Mol Sci. 2021 Sep 14;22(18):9938. doi: 10.3390/ijms22189938 (PMC8471098; doi:10.3390/ijms22189938)
Supplement: Supplementary file 1 [file ijms-22-09938-s001.zip › ijms-1386769-supplementary.pdf]

Supplementary Figure S1: Flow Cytometry detection of CD9, CD81, β1 integrin, α5 integrin, ADAM17 and fibronectin surface molecules on Colo-320 (blue), Colo-320/Cd9 (green), Colo-320/ADAM17-KO (pink).

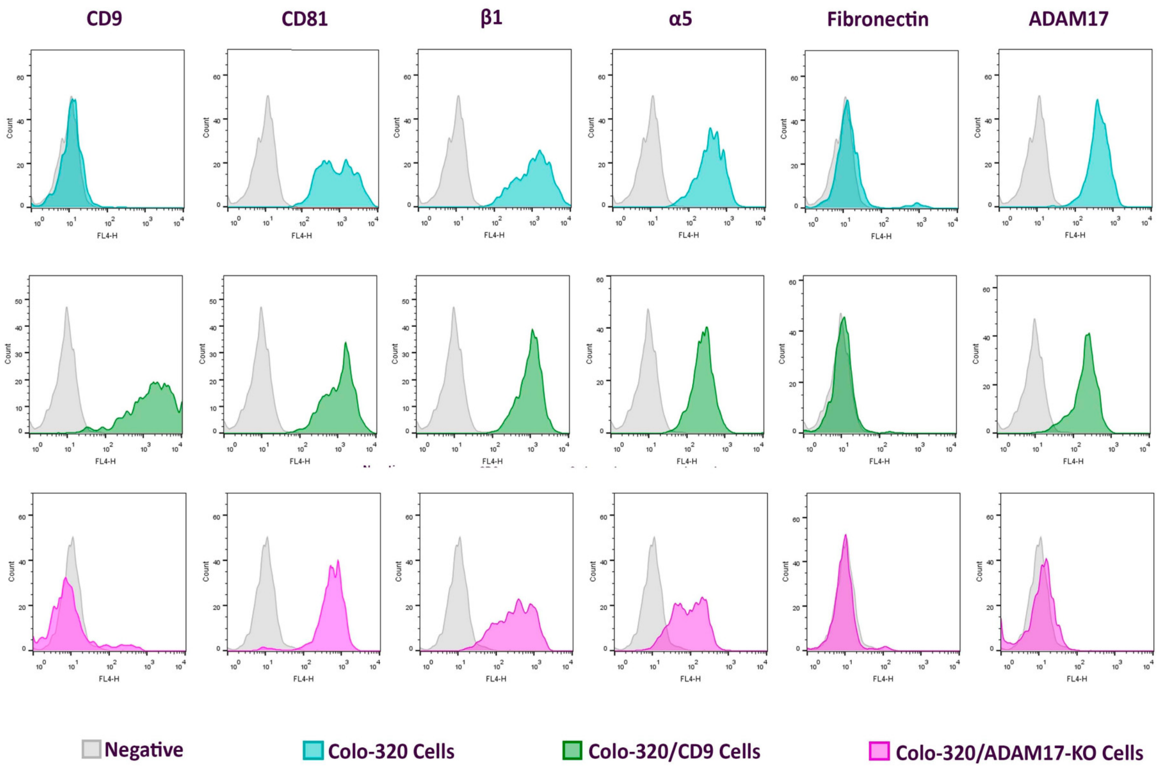

Supplementary Figure S2: (A) Flow Cytometry detection of β1 integrin and fibronectin surface molecules on GMO8402 Fibroblasts. (B) Cell adhesion of Colo-320 cells to immobilized fibronectin in presence of the anti-β1 integrin, anti-α5 integrin and anti-fibronectin mAbs

A.

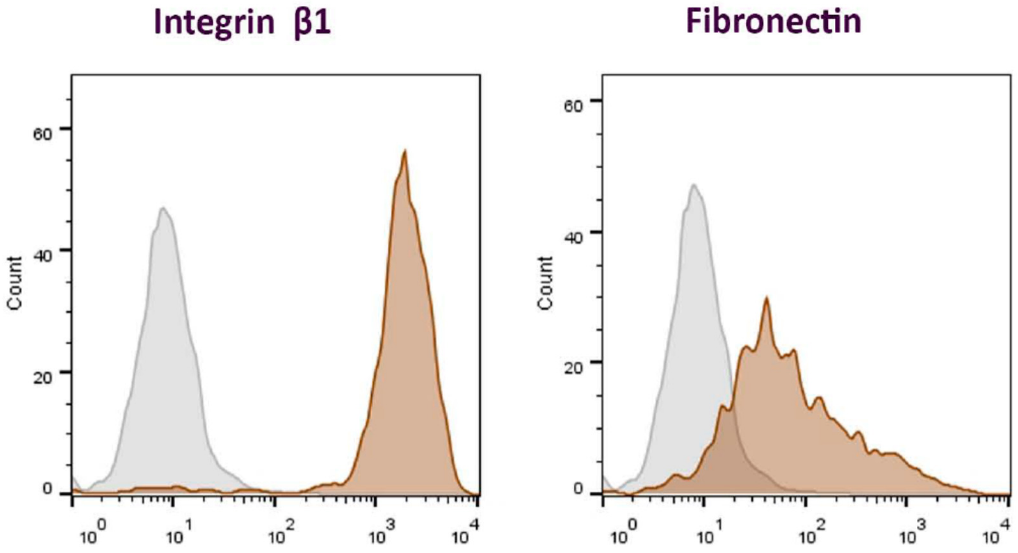

B.

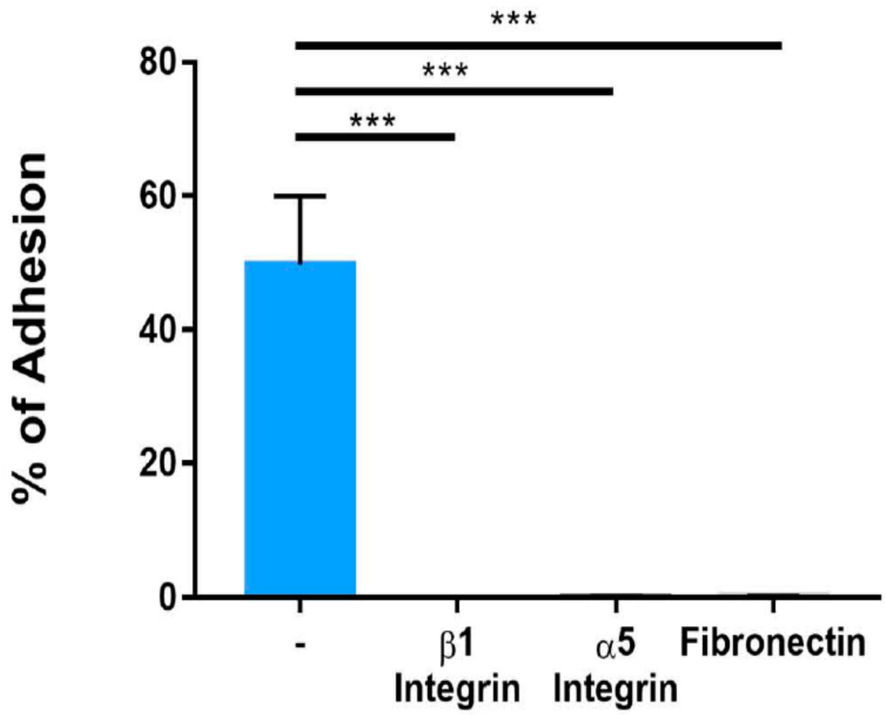

Supplementary Materials and Methods:

Generation of Colo-320/CD9 and Colo-320/ADAM17-KO cell lines from parental Colo-320 cells

To generate the Colo-320/CD9 cell line, 4 x 10^6 parental Colo-320 cells were incubated in 2.5% FCS–RPMI-1640 with the cDNA (20 μg) coding for human CD9 (cloned in the pcDNA3 expression vector). Colo-320 cells were electroporated at 412 V/cm (2 × 10 ms pulses in a 0.4 cm electroporation cuvette) in the ElectroSquarePorator ECM830 (BTX, Holliston, USA), positive clones were selected with G418 (0.8 mg/ml) in the culture medium (1, 2).

To generate the Colo-320/ADAM17-KO cell line, parental Colo-320 cells were transfected with the CRISPR/Cas9 knockout plasmid pX461 encoding GFP and Cas9 nickase and the following sequences to generate the specific single guide RNAs: 5′-CACCGATCTAATATCCAGCAGCATT-3′ and 5′-CACCGTTTTTCTTACCGAATGCTGC-3′. Transfected cells were sorted by flow cytometry based on their positive GFP transient fluorescence and negative expression of ADAM17 and then expanded and rechecked for suppression of ADAM17 expression (2).

References

1. Ovalle, S., Gutierrez-Lopez, M. D., Olmo, N., Turnay, J., Lizarbe, M. A., Majano, P., Molina-Jimenez, F., Lopez-Cabrera, M., Yanez-Mo, M., Sanchez-Madrid, F., and Cabanas, C. (2007) The tetraspanin CD9 inhibits the proliferation and tumorigenicity of human colon carcinoma cells. *International journal of cancer* **121**, 2140-2152

2. Machado-Pineda, Y., Cardenes, B., Reyes, R., Lopez-Martin, S., Toribio, V., Sanchez-Organero, P., Suarez, H., Grotzinger, J., Lorenzen, I., Yanez-Mo, M., and Cabanas, C. (2018) CD9 Controls Integrin alpha5beta1-Mediated Cell Adhesion by Modulating Its Association With the Metalloproteinase ADAM17. *Front Immunol* **9**, 2474
